# Students Assessing Digital News and Misinformation

**DOI:** 10.1007/978-3-030-61841-4_5

**Published:** 2020-10-19

**Authors:** Thomas Nygren, Jenny Wiksten Folkeryd, Caroline Liberg, Mona Guath

**Affiliations:** 8 Leiden Institute of Advanced Computer Science, Leiden University, Leiden, The Netherlands; 9 Leiden Institute of Advanced Computer Science, Leiden University, Leiden, The Netherlands; 10 School of Politics and International Studies, University of Leeds, Leeds, UK; 11 Leiden Institute of Advanced Computer Science, Leiden University, Leiden, The Netherlands; 12 Leiden Institute of Advanced Computer Science, Leiden University, Leiden, The Netherlands; Department of Education, Uppsala University, Uppsala, Sweden

**Keywords:** Media and Information Literacy, Digital literacy, Fake news, Critical thinking

## Abstract

Previous research has highlighted how young people struggle to distinguish news from misinformation. In this study, we investigate how ca. 400 students determine the trustworthiness of false, biased and credible news. We find that students use different strategies depending on what they evaluate. For example, students who fail to debunk a manipulated image often rely on what they see in the image in contrast to students who determine credibility upon what is not in the image. Students finding junk news credible may have special problems separating different kinds of sources. We identify potentials and pitfalls among students important for further investigation, research and a focus on education.

## Introduction

The digitization of society means that news today can be spread quickly and easily, even when it is manipulative and false. The challenge of fake news means that international organizations now increasingly emphasize the importance of digital source criticism. International organizations uphold UNESCO’s so-called Media and Information Literacy (MIL) as an important defense against misinformation^1^ [1]. Media researchers such as Koltay [2] and Carlsson [1] describe MIL as an umbrella term covering other knowledge, skills and attitudes necessary if we are to use new media wisely [1–3]. The ability of people to manage new media can also be described as digital literacy with subgroups such as photo-visual literacy and information literacy [4]. Eshet [4] identifies the ability to read and evaluate digital information (information literacy) as a “survival skill” for citizens in a digital world. Similarly, McGrew et al. [5] emphasize the importance of “civic online reasoning” and “the ability to effectively search for, evaluate, and verify social and political information online”. Even though digitization in society include new aspects, literacy researchers note that the human ability to interact with different forms of artifacts has a very long history [6]. However, the digital dimension involves new potentials and challenges when people search for, access, review, analyze and create information [7–9]. Theories about information literacy that focus primarily on printed text have therefore been expanded also to include multimodal aspects [10].

When it comes to critical and constructive information management, theoretical and empirical investigations have drawn attention to the value of noting who is behind the information, the context of the source, the information in respect of what it alleges, how the information is presented, and why it was created based on underlying purposes [11–14]. Previous research has shown that students have problems with evaluating digital news [5, 15, 16] but have not studied what this is based on more closely.

The purpose of this study is to investigate the way students assess the credibility of digital news and misinformation with regard to whether, and if so how, they express themselves about the source (who), the content (what), the presentation (how) and the underlying purpose (why). In other words, how do they justify their assessments when they in various ways determine the credibility of factual, biased or false information in text and images? Which assessments appear to be more or less successful?.

## Previous Research

Internationally, the poor ability of young people to assess credibility was observed in quantitative studies in the USA [5, 15] and in the latest PISA study [17]. Previous Swedish research has shown that young people have difficulty navigating digital environments [16, 18, 19]. Certain young people may use digital information reflexively, while others easily go astray, maybe due to non-constructive assessments. Studies have shown that people use different cues and heuristics to judge credibility, which can lead to errors. Examples of this include search engine reputation, website design and functionality, previous experiences from websites, and perceived authenticity and expertise in the digital environment [20–23]. The value of reading between the lines and considering different sources are considered to be constructive methods [14, 24]. Studies by professional fact-checkers show that it is crucial to note and evaluate who is behind the information, when and where it was created, the text’s objectivity and bias, underlying objectives and to compare the information with other independent sources [14]. In line with this research, students are recommended to get better at assessing various types of digital news [13, 16, 25]. However, the question is what they base their assessment on.

## Material and Method

This study addresses four tasks used in previous research for evaluating the ability of teenagers to determine the credibility of digital news [16]. Each task contains questions with both fixed response options and open response questions where the students must justify their choice of fixed response options. It is these open text responses that we have analyzed to better understand how the students reason when they assess the credibility of digital news. A total of 483 upper secondary students, age 16-19, took part in the program with a focus on social science (48%), aesthetics (23%), science (16%) and economics (9%). The participants in the study were recruited through their teachers and do not constitute a representative sample. All students agreed to participate anonymously in this study in line with ethical guidelines. Participating students come from different high school programs and they have different backgrounds, but the study’s representativeness is limited to theoretical programs.^2^ The number of students who responded by indicating a fixed response option and those who justified their responses are shown in the result section below.

In line with previous research, the four tasks were designed and found useful for testing the ability of students to identify the source, evaluate evidence and corroborate news [5, 16]. The first task investigated the ability of the students to distinguish news written by journalists to objectively inform from advertising created to manipulate the reader into purchasing something. The second, the ability of the students to assess the validity of the evidence in a task with a manipulated image. In this case, the manipulated image of a smoker’s cheek and mouth was not proof that smoking damages the heart and blood vessels. In the third task, the students had to compare two news reports from a press conference held by the government. A straightforward, objective report from a public service had to be compared with a more biased report from a right-wing populist magazine. Comparing and assessing argumentation was also included in the fourth task where a plastic surgeon’s statements concerning weight reduction were compared to current research into weight reduction. This task investigated the ability of the students to compare different statements about an important health issue where independent research was placed in relation to statements by a plastic surgeon at a company that sells gastric surgery and other forms of plastic surgery.

Our analysis is based on a mixed methods approach where we combine both qualitative and quantitative considerations. The analysis of the student justifications was based on the above-mentioned four dimensions deemed crucial when it comes to critical and constructive digital information management: *who* is behind the information, *what* is the information alleged to contain, *how* the information is designed and *why* it was created on the basis of underlying purposes. Three of the participating researchers in an iterative, multi-step process, carried out the analysis. The researchers switched between analyzing parts of the material (approx. 10% of the student responses in each task) individually and verifying their respective analyses with each other. When a reliable inter-rater consensus was reached (minimum 80%), the researchers analyzed the remaining responses in the tasks based on the established coding schedule (see Appendix A).

In order to evaluate whether the coded categories reflected performance on the respective items we made logistic regressions with fixed response questions as depended variables and the respective coding categories as independent variables for each item. An answer that was included in a category was coded as 1 and otherwise 0. The coefficients for logistic regressions denote the log odds for going from an incorrect to a correct answer on the dependent variable. A negative coefficient denotes a smaller log odds for a correct answer, whereas a positive coefficient represents a larger log odds for a correct answer. We report coefficients and p-values for the significant effects in the text, for a full specification of the models we refer to Table 5, 6, 7 and 8 in Appendix B.

## Results

### Aftonbladet - Distinguishing News from Advertising

This task investigated how students are able to distinguish editorial material from advertising material in an authentic front page from Aftonbladet. The task was formulated as follows: “This is Aftonbladet’s website. The page contains news and advertising. Using the arrows 1 to 5, mark the articles below which you believe to be news. Explain why you think they are news”. The page students had to assess was a screen shot directly from www.aftonbladet.se.

Of the 483 students who answered the question, only 116 (24%) distinguished between news and advertising by identifying the only two alternatives that were news. The remaining students interpreted the other alternatives also as news. Of the 483 students who answered the question, 395 also justified their answers (see Table 1.) Only a few (13 students) considered who the source was.

**Table 1.** Student justifications for their assessments of what is news or advertising

	Answers with comments		Main type of justification							
	N	%	WHO? Source		WHAT? Contents		HOW? Structure		WHY? Function	
Correct	91	23	6	7%	39	43%	45	49%	21	23%
Incorrect	304	77	7	2%	173	57%	138	45%	58	19%
*TOT.*	*395*	*100*	*13*	*9%*	*212*	*100%*	*183*	*95%*	*79*	*42%*

**Note:** Often, student justifications include combinations of these four main inputs. Accordingly, the total number of justifications exceeds the number of participating students.

Students noting how the page was designed, with for instance labels telling readers what is advertising, were significantly better at separating advertising from news than other students [*b* = 3.410, *p* < .001]. 3 They note the design of the texts as a justification for their assessments. This concerns markings of advertising and news and include considerations of headlines, color or position on the page, for instance “one article is clearly placed beneath the heading ‘News’” and “it says news in red and not advertising or weight club”. However, how a page appears may also trick students into believing that everything in the right side column is advertising.

Students who correctly distinguished between news and advertising made comments about how news should be about things that have happened, have a basis in actual circumstances and be based on reviewed information. Some examples from students include “Because this ‘news’ is either proven or can be proved by others” and “Because they inform about facts such as…”. Some students also emphasized that ads can be seen as click baits and an attempt to attract purchases as in the text about the weight club, which is not marked as advertising. Thus in these cases, the content assessed as a combination with its purpose. Another purpose students criticize is that news is written to inform, which a number of students compare to the advertisement’s sales interest: “Because its purpose is information” and “It is information rather than an attempt to sell something”.

Among the 304 students who incorrectly identified news as advertising and vice a versa, we find good arguments, but they lead to incorrect conclusions nevertheless. For example, one student describes how an advertisement for a dietary supplement labeled as advertising, “has nothing to do with advertising” while another student explains in his justification that an advertisement for an energy company is news “because it’s something that concerns us as humans and how we live, and is as such essential”. The contents of advertisements can thus seduce students into believing that it is news, even though it is labeled advertisement. Other students perceive, wrongly, that the advertisements “have nothing to do with products”. Among the students, we also find others who are very critically regard news to be more biased than the advertising. Just under half of these 304 students also seem to have problems identifying which heading or content a highlight on the website refers to. With the argument “it says news where there’s news” and “it says news above news, not advertising” the students failed to identify news, despite its being marked as such. Layouts with “news on the left side” as one student puts it, is also used. One fifth of the students display problems with their ability to see through the purpose of various texts. They express this as e.g. “None of this seems to be trying to sell us anything, but is just a straightforward heading with information” and “because it’s just news and they’re not trying to sell anything”.

### Smoking – Evaluating Evidence

In this task, a manipulated image was used deceptively to show severely injured blood vessels in the cheek of a smoker. 4 The task given to students was formulated as follows: “Smoking may not only cause cancer but also serious damage to the cardiovascular system with an impact on blood vessels and the heart. Can the above picture of Kai Bastard be seen as evidence that such injuries can occur through smoking? Please justify your response”

406 students answered the question. Of them, 307 (76%) noted that the image could not be seen as evidence for injury occurring through smoking, while 99 students (24%) stated that the image could be seen as evidence for the harmful effects of smoking. Of the 406 students, 361 justified their answers (see Table 2).

**Table 2.** Student justifications for assessments of the manipulated image as evidence for the harmful effects of smoking

	Answers with comments		Main type of justification									
	N	%	WHO? Source		WHAT? In the image		WHAT? Outside the image		HOW? Structure		WHY? Function	
No	280	78	10	4%	13	5%	149	53%	154	55%	43	15%
Yes	81	22	1	1%	61	75%	12	15%	12	15%	12	15%
*TOT.*	*361*	*100*	*11*	*5%*	*74*	*80%*	*161*	*68%*	*166*	*70%*	*55*	*30%*

**Note:** Often, student justifications include combinations of these five main inputs. Accordingly, the total number of justifications exceeds the number of participating students.

Few students addressed the issue of the source (who), while many spoke about content (what) and design (how) and around one sixth also raised the purpose of the image (why). Students noting how the design looked manipulated were significantly better than other students were at identifying the image as poor evidence [*b* = 1.930, p < .001]. Of the students who assessed the image as inadequate evidence of the harmful effects of smoking, only ten justified this by the lack of information about the source and the lack of references to other sources.

Just over half of the students mentioned the content, and most of them compared the content of the image with their own knowledge (what; outside the image) and they were significantly better at identifying the image as poor evidence [*b* = 1.736, p < .001]. They wrote such things as “smoking cannot do that. My grandfather smoked almost all his life and had no dark veins in the mouth” and “I have never seen that effect from smoking before”. Several students who assessed the image as poor evidence combined what, how and sometimes also why in their assessments with justifications such as “It’s not an authentic picture; it’s probably been photoshopped because veins in the face never get black through smoking as in the picture” and “it’s been photo shopped; we see people who smoke and they don’t look like this, the image is intended to frighten”. In other words, they placed great importance on how the design of the image seemed to be faked or manipulated in some way. In the assessment concerning how the image appears faked, they also referred to their own digital capabilities in editing images, e.g. “The image can be edited, retouched, photoshopped, counterfeited and so forth” and “because part of my course includes learning Photoshop, I know how easy it is to manipulate a picture”. The students who also assess the purpose saw the image more as a way of frightening people based on a false or exaggerated example.

In contrast students with a focus on what was in the image showed a significant inability to identify the image as manipulated [*b* = −2.892, p < .001]. For example, students noted that “we can see how the blood vessels are black, and because the person in the image is smoking we clearly see that this is the cause” and “we see how the poison that gets into the body spreads throughout the rest of the body”. Also, 12 students identified the image as true and the purpose of it as an attempt to warn people about the actual harmful effects of smoking, e.g. “the image shows how things can look if you smoke too much, which frightens smokers and leads to their cutting down on smoking”.

### New Legislation Against Hate Crimes – Comparing News Without Source Information

The task presented the students with two texts reporting on a government press conference about new legislation against hate crimes. One item (Article A), was a text published the day after the press conference in a right-wing populist newspaper classified as a purveyor of junk news [26]. The other text (Article B) was a direct report from the press conference made by Swedish Radio, a Swedish public service broadcaster. In order to investigate how the students compare and assess texts on the basis of aspects other than the source’s credibility, we removed source information about where the text was published. The task asked the students to indicate which of the articles they “believed to be most credible” and to justify their responses. The response alternatives were (1) Article A, (2) Article B and (3) Neither – they appear to be equally credible.

399 students answered the question. Of these, 86 (22%) pointed to the right-wing populist copy (A) as being most credible, 171 (43%) indicated the copy from Swedish Radio (B) as the most credible, and 146 (36%) assessed the texts as equally credible. Of the above, 275 students justified their answers (see Table 3).

**Table 3.** Student assessments of credibility in news copy about new legislation against hate crimes

	Answers with comments		Main type of justification													
	N	%	WHO? Source		WHO? Primary source		WHO? Secondary source		WHO? Proximity in time		WHAT Contents		HOW? Trends		WHY? Function	
Right-wing populist (A)	67	24	25	37%	4	6%	30	45%	5	7%	4	6%	8	12%	1	1%
Public service (B)	144	52	5	3%	96	67%	4	3%	15	10%	14	10%	41	28%	13	9%
Equally credible	64	23	14	22%	19	30%	22	34%	9	14%	15	23%	11	17%	1	2%
*TOT.*	275	100	44	63%	119	102%	56	82%	29	32%	33	39%	60	58%	15	12%

**Note:** Often, student justifications include combinations of these four main inputs. Accordingly, the total number of justifications exceeds the number of participating students.

In contrast to the two previous tasks, there were many students who assessed credibility by attempting to identify who was behind the information (who). The task was designed such that the students were able to do this in four different ways: (1) which source published the text; (2) whether it was based on first-hand information, or (3) was a rendering of second-hand information, and (4) the writer’s proximity in time in relation to the event.

Because the right-wing populist text referred to Swedish Radio as the source for its biased rendering of the press conference, many students incorrectly identified the source as Swedish Radio and therefore significantly more often considered the text to be credible [*b* = −2.80, *p* < .001]. For example: “It’s Swedish Radio; the other text doesn’t show the source or say who wrote it”. However, five students who assessed text B as the most credible, incorrectly identified the source in A as Swedish Radio. But three of the students balanced their misunderstanding with other considerations, one example of this being “On one hand, Article A is from Swedish Radio, which boosts credibility, but this is easy to fake. Article A seems to be too radical, while B seems calm and collected, which feels more reasonable in a newspaper format when they comment on a new reform”. In addition, among the students who assessed the texts as being equally credible, relatively many incorrectly interpreted the source of Article A to be Swedish Radio.

A significant number of students [*b* = 2.886, *p* < .001] mentioned that reproducing an interview with the minister responsible as the primary source was an important reason for assessing the text from the public service (B) as more credible than the text from the right-wing populist newspaper (A); see Table 4. For example: “It is what the Minister of the Interior said, and they even have a quote of what he said about this, so this article is more credible than the one above”. General references to sources were significantly more common among students who saw the right-wing populist text or both texts as equally credible than those who assessed the public service text as more credible [*b* = − 2.743, *p* < .001].

**Table 4.** Student assessments of credibility in articles about weight loss

	Answers with comments		Main type of justification							
	N	%	WHO? Source		WHAT? Contents		HOW? Structure		WHY? Function	
Research	196	52	138	70%	150	77%	18	9%	27	14%
Surgeon	181	48	160	88%	93	51%	36	20%	9	5%
*TOT.*	*377*	*100*	*298*	*159%*	*243*	*128%*	*54*	*29%*	*36*	*19%*

**Note:** Often, student justifications include combinations of these four main inputs. Accordingly, the total number of justifications exceeds the number of participating students.

The fact that the texts were written the same day or the day after the press conference was also given as the reason for an assessment of credibility. One student, who saw text B as the most credible noted that “Article A is dependent on information in Article B as it was published one day later” in contrast to another student who noted that “the date and time make A more credible as there was more time to gather information”.

In addition, students also noted biases in the use of language in the texts. A significant number of students assessed the public service texts as more credible based upon how the texts were balanced or biased [*b* = 1.402, *p* < .001]. Focusing on the function of the texts, one student gave the example “Article A has completely spun the news to make it appear as if the government will punish those critical of immigration”. Another student found that “A uses language that tries to downplay racism (“racism”) and hate crimes” with reference to the populist text’s use of quotation marks in connection with the word racism. A focus on the purposes behind the information also a significant amount of students identify Article B as more credible [*b* = 2.232, *p* = .00868]. Here we find a few students noting Article B as propaganda or a simple attempt to “getting you to react”.

Among the students who identify text A as more credible, there was also the comment “A is a little more honest about how authoritarian the government is, and it does not try to dress up the oppression of differing opinions with fine words”. Other students perceived text A as more credible as it was “impersonal” and “looks more professional”. Of the students who assessed the sources as equally credible, some saw both texts as equally biased while others saw both texts as equally objective. For example, one student thought that “the people who published the articles just want to create drama” while another felt that “neither seemed biased”.

### Weight Loss - Comparing Complex Articles on Health

In this task, information about ways to lose weight were compared with each other. Statements from a surgeon who performed weight loss procedures published in a morning newspaper (Article A), were compared to a recent study from a publicly funded, top ranking university published by a weekly paper with a section that focused on health issues (Article B). The task was worded as follows “Is Article A or B more credible as a source of information about weight loss? Please justify your choice of A or B. Why is this article more credible?”

Of the 420 students who answered the question, 222 (53%) saw the research-based article as more credible, while 198 (47%) judged the other article to be more credible. Of the 420 students, 377 justified their answers (see Table 4).

In contrast to the three previous tasks, a different pattern can be seen here with most justifications relating to who and what. The students who assessed the article that presented recent research as most credible pointed significantly more at the content (what?) [*b* = 0.809, p < .001] and the purpose (why?) behind the information [*b* = 1.107, p = .008]. They point out that the article contains research in the form of a study carried out at the university and also includes statistical data. The justifications they provide regarding this are e.g. “it sounds reasonable and has a lot of figures and studies which make me think that it’s the more credible” and “because it’s a fact that exercise reduces weight and going on a diet helps weight loss, but diet alone doesn’t work”. Some of the students also compare the two articles as in “A is only about a person’s thoughts while B has a carried out a study that supports it (even if it is a small study) and explains why things are the way they are instead of just saying it is what it is”. Students commenting the purpose of the articles finds that the interview with the doctor is not credible “because he wants more people to have procedures so he can earn more money”, i.e. he wants to advertise his procedures. Not many of the students raise the design of Article B as credible. The comments made by those who do, point out that the article about research has more links to sources, is more nuanced, looks more professional and is thus more credible.

In contrast, those who assessed the article based on the surgeon’s statements as being more credible did so in most cases based on an assessment of the source (who?). The fact that the information was published in an established morning newspaper was given by a significant number students as the main reason for assessing the article as credible [*b* = −1.073, *p* < .001]. In addition, a significant number of the students [*b* = −0.806, *p* = .0131] also based this judgement upon the credible design (how?) of the morning newspaper, for instance in comparison to how “the other article reads more like a blog”.

## Conclusions

This study shows that youths often find it difficult to determine credibility when faced with different types of digital news and misinformation. The results suggest that different strategies are necessary for navigating credible, biased or false news.

Several previous studies have noted that students find it difficult to distinguish news from advertising [5, 15, 16, 25]. Our findings in this study show that this may depend on a number of factors. In comparison with other students, those who succeed best are able more often to identify how advertising is labeled and they are able to navigate the design of the page. However, it is also difficult for many students to know how to interpret advertising labels. Labeling above an advertisement, aimed at helping the reader see that something is an advertisement, may be interpreted as applying to the text above rather than the ad. Students can also be tricked by the location of the news on the web page. Items placed to the right or left are easily interpreted as advertisements, probably due to the experience of ads following customers in the margins on various web pages.

Examinations carried out by students of a manipulated image purporting to be evidence of the harm caused by smoking show other assessment patterns. In this case, many students make relevant assessments based not on what is presented in the image but on their own experience and knowledge and how easy it is to manipulate images using e.g. Photoshop. On the other hand, students can be misled by a focus on the strong content in the image. The students who arrive at inaccurate assessments are often convinced of the image’s authenticity and they regards its symbolic content as facts. The students’ previous knowledge and familiarity with handling manipulated images is thus at the heart of their ability to assess this type of task.

When it comes to student assessment of more objective news versus biased news regarding new legislation against hate crimes, it is primarily the ability to distinguish first-hand information from second-hand information that would appear to be key. In this task, critical aspects that focus on different types of sources, primary and secondary, appear to lead in the right direction. Students who consider *how* the information can be biased through the use of language and identified the underlying purpose of the right-wing populist news also found the text from public service to be more credible.

What made students assess the right-wing populist news as more credible was often incorrect identification of who the source was, their problematic interpretation of proximity in time and their treatment of sources in a non-specific manner. Students who referred to sources without distinguishing between them often gave equal credibility to both texts or greater credibility to the right-wing populist text. Thus, *who* was behind the first-hand information and *how* texts can be biased were key in managing this task.

When comparing the texts regarding weight loss, it was mainly students who considered the content (what) and the text’s function and potential underlying purpose (why) that determined the researched-based article to be the most credible. Those who assessed the text on the premise of the plastic surgeon’s statements did so to a greater extent based on how the article looked and the fact that it was published in an established morning newspaper (who).

## Concluding Discussion

There seems to be a number of reasons why it is difficult for young people to distinguish news from advertising. It may in part be due to a lack of experience in reading the newspaper we used in the study, despite the fact that previous research found it to be a popular source of news among many young people in Sweden [27]. That so many missed or misunderstood advertising labels may be due to a lack of experience or attention. It was apparent in the student assessments that labeling, color and location may not only act as an aid, but can also easily be misunderstood. The fact that information in the margins can be interpreted as advertising indicates a heuristic approach that can easily lead to errors. Important information can be missed and new techniques for inserting advertisements in a more central location in digital environments may lead to misunderstandings. The example of this website also shows the real issues with the blurred line between news and advertising. In journalism, it is crucial to distinguish between content produced with and without the influence of direct market interests [28]. But it is obviously difficult for the reader in digital environments to distinguish between them, especially when the material is written by journalists and in a form that is very similar to news produced to inform and not to manipulate [29]. It is clear that many young people need to be better at distinguishing news from advertising. It remains to be seen how teaching can help here as previous attempts at teaching students in this matter has had very limited effect [25]. The fact that many students do not explicitly note the source would seem to be a problem, even if it was not a significant factor in our study. Perhaps this is the reason why so few students succeed in distinguishing news. Thus, the design of teaching methods that help more students reflect over who a source is may be worth exploring in future research. Our findings indicate that it could be constructive for teachers to show and discuss the design of news pages, since many students have difficulty understanding labeling and layout. A lack of practice and experience in handling news and advertising may need to be weighed up in teaching.

The task with the manipulated image of the smoker highlights the importance of possessing good subject knowledge. Those who compared the content of the image with information outside the image were able to determine its credibility on this basis. Other students were caught up in the deceptive design of the image. This may also depend on emotional aspects where strong visual messages can be misleading. With regard to evaluating the manipulated image, personal experience from having worked with image manipulation also appears to be an advantage much in line with previous research [30]. As regards education, this not only shows the importance of schools teaching about actual factual circumstances, but also allowing students to test new image manipulation technologies.

Students who found the right-wing populist text to be more credible than the text from Swedish Radio were in many cases mistaken about who the source was. The students who incorrectly believed that text A came from Swedish Radio often chose to name it as being more credible. Thus, the main reason for choosing the right-wing populist text was the students’ misinterpretation of who was behind the text. This demonstrates the importance of the ability to correctly identify the source. It can be difficult, especially in digital environments to determine who is behind the information when fake accounts are created to disseminate misleading information. The false account phenomenon has been noted as a serious threat to democracy [31], especially as disinformation can be used to reinforce divisions in society and spread discontent and suspicion.

This especially highlights the importance of the ability to distinguish between primary sources and secondary sources. Those who understood the value of information on the basis of a primary source found success, while others who were more careless and considered different sources as equivalent, failed. Thus not being fooled by references to more credible sources puts students on the right track. The fact that external websites with misleading information pages refer to other sources with high credibility has already been identified as a manipulative strategy that can even mislead highly educated people [14]. Another problem identified is where students feel that indirect reports can be seen as better. Thus, source-critical knowledge about proximity in time and space is important and must be reinforced.

Our findings also shows that it can be constructive to consider how texts can be written to inform objectively or to manipulate. Students who focused on the underlying purpose about why a text was written also succeeded in navigating the text in a critical, constructive manner. The fact that there were relatively few students who based their assessments on how and why the texts were created is a challenge for future research and teaching. Seeing and interpreting trends does not seem to form a natural part of the assessments of many students. The study also shows that there is a risk that texts are interpreted as equally factual or biased, despite their possessing distinct differences. There is clearly a need for more training in calling the author into question and the ability to recognize what is worth trusting.

The difficulty in choosing between conflicting information was also clear in the fourth task. Previous research has underscored the importance of source credibility [32] and students identifying the morning paper as credible missed the fact that the primary source in the article was not as credible as research from a university. Self-assured statements from a doctor in an established morning newspaper were deemed by many to be more credible than less overconfident research outcomes from a recent minor study published in a paper with less credibility. Thus, layers of source credibility made it hard for students to navigate this task. In spite of this problem, the majority of students in this case assessed the article with references to research as the most credible. Students who named the article about research as being the most credible focused on the more objective content of the article, which put across what the complex facts about weight loss the research had arrived at, and the underlying purpose of a plastic surgeon in private practice. This task also showed the need to get more students to consider why the information was created and communicated in the manner chosen. In the case of issues about health advice, there are obvious challenges as advertisements for dietary supplements are interpreted as news, while research can be regarded as less credible than plastic surgeons. Dietary and health advice is evidently difficult to assess. In a digital world with many affluent stakeholders [33], young people need guidance about e.g. food and cosmetic surgery. They need the ability to evaluate available information. We need to study how this can be done in critical, constructive ways in more detail in future research.

What is clear from this study is that the issues raised as crucial in terms of information handling – who, what, how and why – are useful for assessing digital news. However, it is evident that different types of news and misinformation require different types of assessments. In education, we need to find ways to support multiple ways of assessing credibility, while also understanding the risk that focusing on the right thing may still lead to the wrong conclusion.

## Limitations

Questionnaires and tests were completed in a classroom environment, and student vigilance regarding manipulations may therefore have been higher than otherwise when encountering credible, biased and fake news on the Internet. We did not ask students about their habits and experiences of smoking and diets, which may have affected their motivated reasoning.

## Appendix A Coding Schedule

Aftonbladet

1. Who? – source – identifies who is behind the information.
2. What? – content – describe what the information is about.
3. How? – structure – describes design.
4. Why? – function – the purpose is to sell something or to objectively inform.

Smoking

1. Who? – source – identifies who is behind the information,
2. What? – content – describes the knowledge present in the image.
3. What? – content – compares the image to knowledge outside the image.
4. How? – structure – describes design e.g. Fake, manipulated, metaphorical image, symbolic
5. Why? – function – the purpose is to objectively inform or manipulate.

Hate crimes

1. Who? – source – identifies (incorrectly) Swedish radio as the source in article A.
2. Who? – source – identifies the source in B as first-hand (primary source).
3. Who? – source – refers to a more general source or secondhand information.
4. Who? – source – identifies the articles as having different publishing dates.
5. What? – content – describes what the information is about.
6. How? – structure – neutral or biased use of language.
7. Why? – function – purpose is to objectively inform or manipulate.

Weight loss

1. Who? – source – identifies who is behind the information.
2. What? – content – describes what the information is about.
3. How? – structure – describes design.
4. Why? – function – the purpose is either to sell something or to objectively inform

## Appendix B

**Table 5.** *Estimates of Best Fitting Logistic Regression Model for Correct/Incorrect Answer on Hate crime, with Coefficients Denoting the Log Odds of Answering Correct when Classified in or Not in one of the Coding Categories Who 1, Who 2, Who 3, Who 4, What, How and, Why.*

Coefficent	Estimate	Std. error	z value	Pr(>|z|)
(Intercept)	−1.005	.184	−5.450	<.001
Hate crime who 1	−2.680	.579	−4.633	<.001
Hate crime who 2	2.886	.338	8.544	<.001
Hate crime who 3	−2.743	.633	−4.331	<.001
Hate crime who 4	0.121	.521	0.233	.816
Hate crime what	0.642	.432	1.486	.137
Hate crime how	1.402	.384	3.649	<.001
Hate crime why	2.232	.851	2.624	0.00868

*Note:* Residual deviance: 349 on 391° of freedom. AIC = 365

**Table 6.** *Estimates of Best Fitting Logistic Regression Model for Correct/Incorrect Answer Aftonbladet with Coefficients Denoting the Log Odds of Answering Correct when Classified or Not in one of the Coding Categories Who, What, Why and, How*

Coefficent	Estimate	Std. Error	z value	Pr(>|z|)
(Intercept)	−4.159	0.621	−6.698	<.001
Aftonbladet who	−0.548	1.126	−0.487	.627
Aftonbladet what	−0.809	0.452	−1.790	0.0735
Aftonbladet why	−0.104	0.602	−0.173	0.863
Aftonbladet how	3.410	0.622	5.484	<.001***

*Note:* Residual deviance: 249 on 478° of freedom. AIC = 259

**Table 7.** *Estimates of Best Fitting Logistic Regression Model for Correct/Incorrect Answer on Weight Loss, with Coefficients Denoting the Log Odds of Answering Correct when Classified or Not in one of the Coding Categories Who, What, How and, Why.*

Coefficient	Estimate	Std. error	z value	Pr(>|z|)
(Intercept)	0.431	.232	1.862	0.0626
Weight loss who	−1.073	.240	−4.478	<.001
Weight loss what	0.809	.215	3.765	<.001
Weight loss how	−0.806	.325	−2.480	0.0131
Weight loss why	1.107	.417	2.654	0.00797

*Note:* Residual deviance: 527 on 414° of freedom. AIC= 537

**Table 8.** *Estimates of Best Fitting Logistic Regression Model for Correct/Incorrect Answer on Smoking, with Coefficients Denoting the Log Odds of Answering Correct when Classified or Not in one of the Coding Categories Who, What 1, What 2, How and, Why.*

Coefficient	Estimate	Std. error	z value	Pr(>|z|)
(Intercept)	0.667	0.270	2.469	.0135
Smoking who	2.529	1.291	1.959	.0501
Smoking what 1	−2.892	0.393	−7.367	<.001
Smoking what 2	1.736	0.392	4.426	<.001
Smoking how	1.930	0.398	4.854	<.001
Smoking why	0.357	0.484	0.738	0.461

*Note:* Residual deviance: 254 on 397° of freedom. AIC = 266

## Footnotes

1 In this article, we define misinformation as inaccurate, manipulative or false information, including disinformation, which is deliberately designed to mislead people.

2 In Sweden, 2017, approx 30% studied social science, 11% aesthetic, 22% science and 20% economics (SiRiS). Our study does not include students in vocational training. One third (34%) of the students stated that they speak a different language at home. Of the respondents, 60% described themselves as girls, 35% as boys, 3% as other identity and 2% chose not to present their gender.

3 The same result was obtained if number of correct items was regressed on the categories: there was a larger log count [*b* = 0.331, p < .001] for those whose comment was categorized as “how”. Mean number of correct was 4.008 (*SD* = 1.254).

4 Image source: https://www.vice.com/en_uk/article/yp54bw/kai-bastard-photo-manipulations
